# *UCHL1* Expression in Colorectal Cancer: Clinicopathological Significance, Prognostic Value, and Implications for Immunotherapy Response

**DOI:** 10.3390/biomedicines14091924

**Published:** 2026-08-27

**Authors:** Jiming Gu, Suhua Xia, Tongguo Shi, Dongming Zhu

**Affiliations:** 1 Department of General Surgery, The First Affiliated Hospital of Soochow University, Suzhou 215000, China; jiminggu2026@163.com; 2 Department of Oncology, The First Affiliated Hospital of Soochow University, Suzhou 215000, China; xiasuhua@suda.edu.cn; 3 Jiangsu Institute of Clinical Immunology, The First Affiliated Hospital of Soochow University, Suzhou 215000, China

**Keywords:** colorectal cancer, UCHL1, TCGA, prognosis, immune

## Abstract

**Background/Objectives**: Ubiquitin C-terminal hydrolase L1 (*UCHL1*) exhibits context-dependent roles in various cancers, but its clinical significance and biological functions specifically in colorectal cancer (CRC) remain incompletely understood. **Methods**: We systematically evaluated UCHL1 expression, clinicopathological associations, prognostic value, and immunological role in CRC using multiple public databases (TCGA, GTEx, UALCAN, GEPIA2, GSCA, and ENCORI) combined with immunohistochemical validation on a tissue microarray containing 80 paired CRC and adjacent normal tissues. Functional enrichment was assessed using ssGSEA, and immune cell infiltration was analyzed using the immunedeconv R package. The immunotherapy response was evaluated using the TIDE algorithm. **Results**: *UCHL1* mRNA and protein levels were significantly downregulated in CRC tissues compared with normal tissues. Paradoxically, high *UCHL1* expression was significantly associated with advanced T stage, N stage, TNM stage, and poor overall and disease-free survival. ssGSEA revealed positive associations with multiple aspects of oncogenic pathways, including tumor inflammation signature, tumor proliferation signature, epithelial–mesenchymal transition markers, extracellular matrix-related genes, angiogenesis, apoptosis, and G2M checkpoint regulation. Notably, *UCHL1* expression was positively correlated with computationally estimated infiltration of macrophages, CD4^+^ T cells, and CD8^+^ T cells, and with elevated expression of immune checkpoint genes, as well as higher TIDE scores. These correlative findings suggest a potential association with immunotherapy-related pathways that warrants further investigation. **Conclusions**: *UCHL1* is downregulated in CRC, but its elevated expression is associated with aggressive disease and poor prognosis. It is implicated in multiple oncogenic pathways and may contribute to an immunosuppressive tumor microenvironment, suggesting its potential as a candidate prognostic biomarker that warrants further functional investigation.

## 1. Introduction

Colorectal cancer (CRC) is highly malignant and associated with substantial global mortality [1]. In 2022, CRC accounted for approximately 1.9 million new cases and 90,000 deaths, representing one of the most prevalent malignancies worldwide [2]. Although advancements in screening and adjuvant therapy have improved the five-year overall survival rate for localized CRC in recent years, the prognosis for advanced or metastatic disease remains poor [3]. The absence of distinct early clinical manifestations often leads to diagnosis at an advanced stage, with approximately 20% of patients presenting with distant metastases at initial diagnosis [4,5,6]. Therefore, identifying diagnostic biomarkers and developing novel therapeutic strategies are imperative to improving clinical outcomes in patients with CRC.

Ubiquitin C-terminal Hydrolase L1 (*UCHL1*), also known as PGP9.5 or PARK5, is a multifunctional deubiquitinating enzyme that participates in the ubiquitin-mediated protein degradation pathway, exhibiting effects such as deubiquitination, ligation, and hydrolysis [7]. In cancer pathogenesis, UCHL1 plays context-dependent and often opposing roles, acting either as a tumor suppressor or an oncogene depending on the malignancy. For instance, it promotes tumorigenesis in neuroendocrine carcinomas, triple-negative breast cancer, and gastric cancer [8,9,10], whereas it suppresses tumor development in nasopharyngeal carcinoma [11]. In CRC, UCHL1 has been reported to function as an oncogene by activating the β-catenin/TCF pathway through its deubiquitinating activity [12]. Additionally, *UCHL1* promoter methylation has been identified as a potential marker for metastatic CRC [13], and its level of expression correlates with lymph node metastasis in colorectal carcinoma [14]. Despite these emerging insights, the diagnostic value of UCHL1 in CRC remains unclear.

In this study, we aimed to comprehensively evaluate the expression, clinicopathological associations, and prognostic significance of *UCHL1* in CRC through a bioinformatics analysis integrating multiple public mRNA databases. Further analysis revealed that elevated UCHL1 protein levels in CRC tissues correlate with N and AJCC stages. Potential biological pathways regulated by *UCHL1* during CRC progression were subsequently identified. Additionally, the impact of *UCHL1* on immune infiltration and immunotherapy response was assessed.

## 2. Materials and Methods

### 2.1. Analysis of UCHL1 mRNA Expression

*UCHL1* mRNA expression in colorectal cancer and normal tissues was examined using four databases—UALCAN (https://ualcan.path.uab.edu/, accessed on 24 March 2026), GEPIA2 (http://gepia2.cancer-pku.cn/#index, accessed on 24 March 2026), GSCA (https://guolab.wchscu.cn/GSCA/#/, accessed on 24 March 2026), and ENCORI (https://rnasysu.com/encori/, accessed on 24 March 2026). TCGA-derived STAR-counts data, together with corresponding clinical information, were downloaded from the GDC data portal (https://portal.gdc.cancer.gov, accessed on 24 March 2026). The raw read counts were converted to TPM values and normalized via log2(TPM+1) transformation, yielding 620 eligible CRC samples after filtering. Normal tissue expression profiles were obtained from the GTEx project (V8 release; https://gtexportal.org/home/datasets, accessed on 24 March 2026). All statistical comparisons of *UCHL1* mRNA abundance between CRC-TCGA and GTEx samples were executed in R (version 4.0.3) [15]. The beta value, which indicates the DNA methylation level at the *UCHL1* promoter, was analyzed using the UALCAN database (https://ualcan.path.uab.edu/, accessed on 19 August 2026).

### 2.2. Association Between UCHL1 mRNA Expression and Clinicopathological Parameters

Based on the 620 CRC samples obtained from TCGA (https://portal.gdc.cancer.gov, accessed on 24 March 2026), patients were stratified into two groups according to the median *UCHL1* expression level, yielding 311 cases with high expression and 309 with low expression. Associations between *UCHL1* expression and clinicopathological variables—including age, gender, race, T stage, N stage, M stage, TNM stage, and new tumor event—were evaluated using the chi-squared test. All statistical analyses were performed using R software (version 4.0.3) [16].

### 2.3. Survival Prognosis Analysis

To evaluate the prognostic value of *UCHL1* in CRC, we utilized three independent datasets. The GEPIA2 database, which integrates TCGA and GTEx expression data, was used to evaluate overall survival (OS)—defined as the time from diagnosis to death from any cause—and disease-free survival (DFS)—defined as the time from treatment completion to disease recurrence or death. For this analysis, patients were stratified into groups based on quartiles of *UCHL1* expression. Additionally, the Kaplan–Meier Plotter database (https://kmplot.com/analysis/, accessed on 24 March 2026), which integrates gene expression and clinical data from GEO, TCGA, and EGA, was used to assess OS (time from diagnosis to death) and DFS (time from treatment completion to recurrence). Finally, the TCGA cohort (*n* = 620) was analyzed, for which RNA-seq data and corresponding clinical information were obtained from the TCGA GDC data portal (https://portal.gdc.cancer.gov, accessed on 24 March 2026); only samples with complete RNA-seq data, non-missing clinical information (including survival time, T/N/M stage, and TNM stage), and non-zero survival time were included, with OS defined using the TCGA “OS” attribute (time from diagnosis to death) and DFS defined as time from treatment completion to recurrence or death. For all three datasets, Kaplan–Meier curves were generated, and log-rank tests were performed to compare survival differences between the high- and low-*UCHL1* expression groups [17,18,19].

### 2.4. Function Analysis

For single-sample gene set enrichment analysis (ssGSEA), we applied the same TCGA CRC dataset described above (https://portal.gdc.cancer.gov, accessed on 24 March 2026). Predefined pathway gene sets were assembled and processed using the GSVA package in R (version 4.0.3), with the method parameter set to “ssgsea” [20,21].

### 2.5. Immune Correlation Analysis

To investigate the relationship between *UCHL1* expression and immune cell infiltration in CRC samples from the TCGA database (https://portal.gdc.cancer.gov, accessed on 24 March 2026), immune scores were assessed using the immunedeconv R package (version 2.1.2), which integrates four state-of-the-art algorithms: QUANTISEQ, TIMER, EPIC, and MCPCOUNTER. These algorithms have been systematically benchmarked, each offering distinct advantages in deconvoluting immune cell fractions from bulk transcriptomic data. All statistical analyses were conducted using R software (version 4.1.3; R Foundation for Statistical Computing, Vienna, Austria, 2022), with the correlation analyses and visualizations performed using the ggClusterNet R package (version 2.00) [22].

In addition, Spearman’s correlation analysis was performed to assess the relationship between *UCHL1* and immune cells (B cells, CD4^+^ T cells, CD8^+^ T cells, macrophages, neutrophils, and myeloid dendritic cells) in CRC using TCGA data (*n* = 620) [23].

### 2.6. Relationship Between UCHL1 and Immune Checkpoint Gene Expression

To further explore the relationship between *UCHL1* expression and immune checkpoint regulation, the CRC samples from the TCGA database (https://portal.gdc.cancer.gov, accessed on 24 March 2026) that were dichotomized into high- and low-expression groups based on the median *UCHL1* expression level (high expression, *n* = 311; low expression, *n* = 309) were used. The expression levels of immune checkpoint-associated genes, including *CD274*, *CTLA4*, *HAVCR2*, *ITPRIPL1*, *LAG3*, *PDCD1*, *PDCD1LG2*, *SIGLEC15*, *TIGIT*, and *IGSF8*, were compared between the two groups [24,25]. Moreover, the Tumor Immune Dysfunction and Exclusion (TIDE) algorithm was employed to predict immunotherapy response for each group. Statistical analyses were performed using R software (version 4.0.3) [26].

### 2.7. Human Samples

A CRC tissue microarray (TMA) was obtained from Hunan Aifang Biotechnology Co., Ltd. (Changsha, China), accompanied by corresponding follow-up data. The TMA consisted of 80 paired samples of CRC and adjacent normal tissues (NATs); the patient characteristics are summarized in Table 1. Ethical approval for this study was granted by the Medical Ethics Committee of the First Affiliated Hospital of Soochow University (approval no. 2021-327). Informed consent was obtained from all subjects involved in the study.

### 2.8. Immunohistochemistry

Immunohistochemical staining was conducted on 4 μm serial sections of paraffin-embedded tissue specimens, following a previously established protocol [27]. After deparaffinization and rehydration, antigen retrieval was performed by microwave heating in citrate buffer (pH 6.0). Non-specific binding sites were blocked by incubating the sections with 1% bovine serum albumin (BSA) in PBS for 30 min at ambient temperature. The sections were subsequently incubated overnight at 4 °C with a rabbit polyclonal anti-UCHL1 primary antibody (Proteintech Group, Inc. (Wuhan, China); cat. no. 14730-1-AP) at a 1:200 dilution. Following washing, the sections were treated with a biotinylated secondary antibody (Boster Biological Technology Co., Ltd. (Wuhan, China); cat. no. SA1020; 1:500) and then with HRP-conjugated streptavidin from the same kit. Immunoreactivity was visualized using a DAB+ chromogen kit (Agilent Technologies, Inc., Santa Clara, CA, USA), and the sections were counterstained with hematoxylin. The IHC score was determined by multiplying the staining intensity (negative, 0; mild, 1; moderate, 2; and strong, 3) by the percentage of the stained area (0%, 0; 1–25%, 1; 26–50%, 2; 51–75%, 3; and 76–100%, 4). All sections were evaluated independently by two experienced pathologists who were blinded to all clinicopathological information, including patient identity, disease stage, and survival outcomes. For subsequent clinicopathological and prognostic analyses, CRC samples were dichotomized into high- and low-UCHL1 expression groups using the median IHC score of the 80 paired samples as the cutoff (IHC score > 3 as high expression).

### 2.9. Statistical Analysis

Data analysis and visualization were performed using R software (version 4.0.3) and GraphPad Prism 8.0 (Dotmatics, San Diego, USA). For comparisons between the two groups, paired *t*-tests, unpaired *t*-tests, one-way ANOVA, or Wilcoxon rank-sum tests were applied depending on data distribution and experimental design. For parametric comparisons using *t*-tests, data are expressed as mean ± standard deviation (SD). For non-parametric comparisons using Wilcoxon rank-sum tests, data are expressed as medians with interquartile ranges. Associations between *UCHL1* expression and clinicopathological features were evaluated using Fisher’s exact test or the chi-squared test, as appropriate. Spearman’s rank or Pearson’s correlation analysis was conducted to examine the relationship between *UCHL1* expression and immune cell infiltration in CRC using TCGA data (*n* = 620). *p* < 0.05 was considered statistically significant.

## 3. Results

### 3.1. Expression and Association of UCHL1 with Clinicopathological Variables in CRC Patients

**Figure 1:**
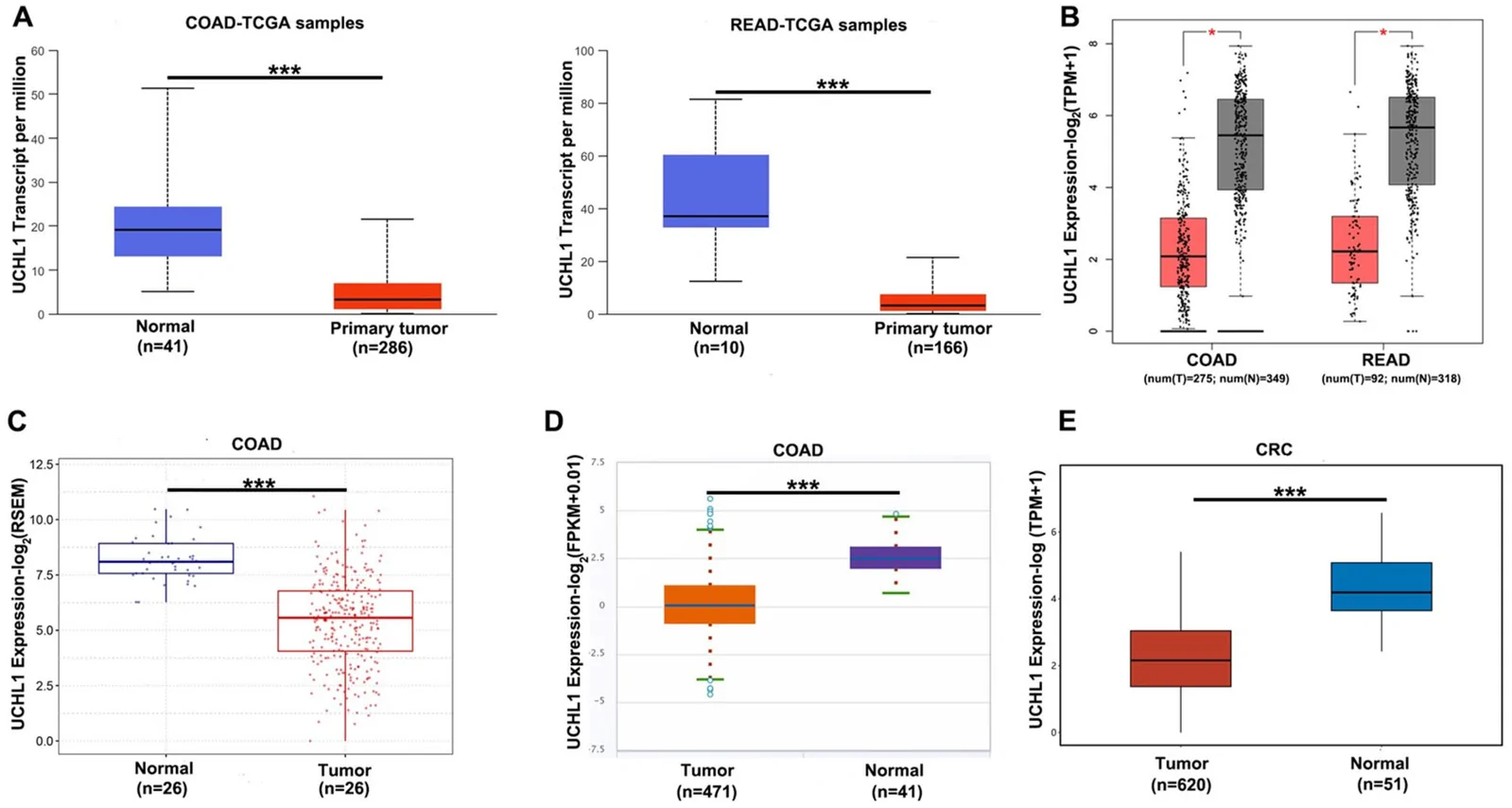
The mRNA expression level of *UCHL1* in CRC and healthy tissues: (**A**–**D**) The mRNA expression of *UCHL1* in CRC and normal tissues in the UALCAN (**A**), GEPIA 2.0 (**B**), GSCA (**C**), and ENCORI (**D**) databases. (**E**) The mRNA levels of *UCHL1* in CRC and normal tissues from TCGA databases. Statistical significance was assessed using an unpaired *t*-test (**A**), one-way ANOVA (**B**), paired *t*-test (**C**), and Wilcoxon rank-sum test (**D**,**E**). * *p* < 0.05; *** *p* < 0.001.

To investigate the expression of *UCHL1* in colorectal cancer (CRC) tissue samples, we utilized multiple database visualization platforms, including UALCAN, GEPIA2, GSCA, and ENCORI. The results showed that *UCHL1* mRNA levels were lower in the CRC tissues than in normal tissues (Figure 1A–D). In addition, we downloaded STAR-counts data and corresponding clinical information for CRC from the TCGA database to further analyze *UCHL1* mRNA expression levels. As shown in Figure 1E, UCHL1 mRNA levels were significantly decreased in CRC tissues compared with normal tissues.

**Figure 2:**
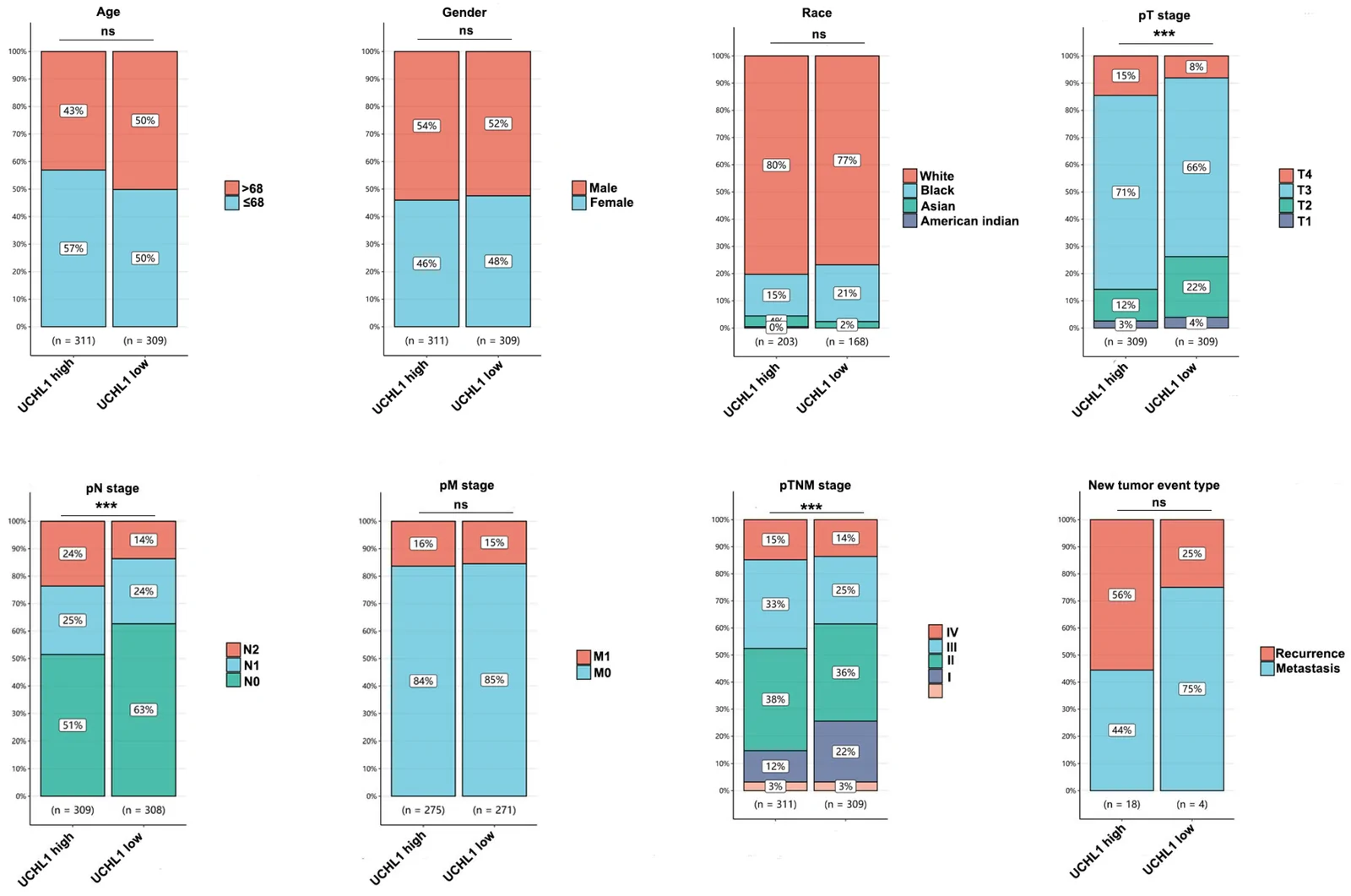
The distribution of clinical features in different CRC groups from the TCGA database. The significance (*p*-value) between different groups is analyzed through the chi-squared test. *** *p* < 0.001; ns, no significant change.

Subsequently, we analyzed *UCHL1* expression in 620 CRC samples from the TCGA dataset. As shown in Figure 2, *UCHL1* expression was significantly associated with T, N, and TNM stage. In contrast, no significant associations were observed with age, gender, race, M stage, or new tumor event type.

### 3.2. Prognostic Value of UCHL1 in CRC

**Figure 3:**
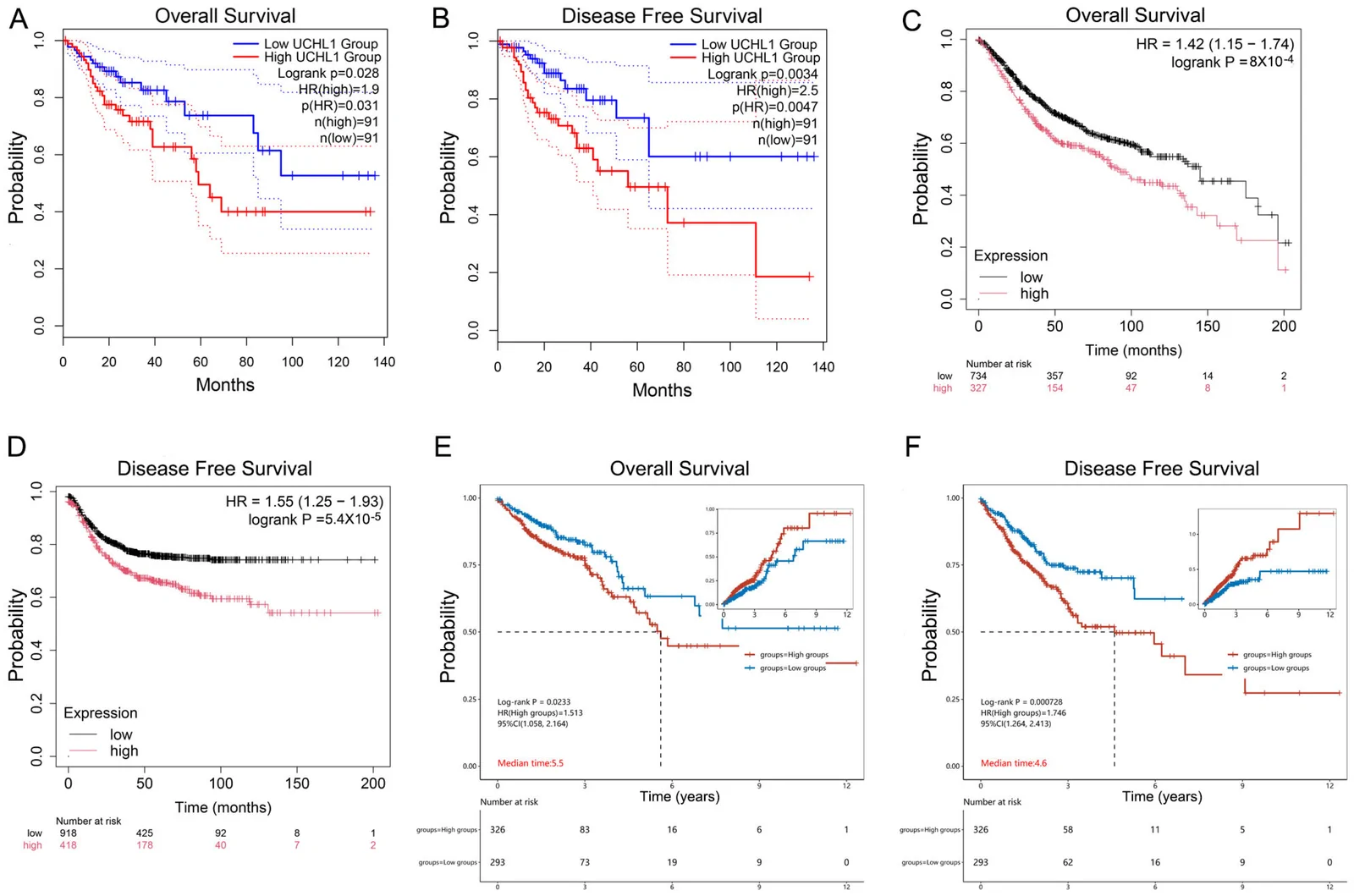
Prognostic value of *UCHL1* in patients with CRC: (**A**,**B**) The relationship between *UCHL1* expression and the overall survival (**A**) and disease-free survival (**B**) of patients with CRC was analyzed using the GEPIA 2.0 database. (**C**,**D**) The relationship between *UCHL1* expression and the overall survival (**C**) and disease-free survival (**D**) of patients with CRC was analyzed using the Kaplan–Meier Plotter database. (**E**,**F**) Kaplan–Meier overall survival (**E**) and disease-free survival (**F**) analyses of *UCHL1* using the TCGA cohort. In all analyses, patients were stratified by *UCHL1* expression, and survival differences were evaluated using the log-rank test.

To investigate the prognostic value of *UCHL1* in CRC, we utilized the GEPIA and Kaplan–Meier Plotter databases. As shown in Figure 3A–D, patients with high *UCHL1* expression exhibited lower overall and disease-free survival than those with low *UCHL1* expression. Furthermore, we validated these findings using the TCGA database, which also indicated that high *UCHL1* expression was associated with a poor prognosis.

### 3.3. The Potential Biological Role of UCHL1 in CRC

**Figure 4:**
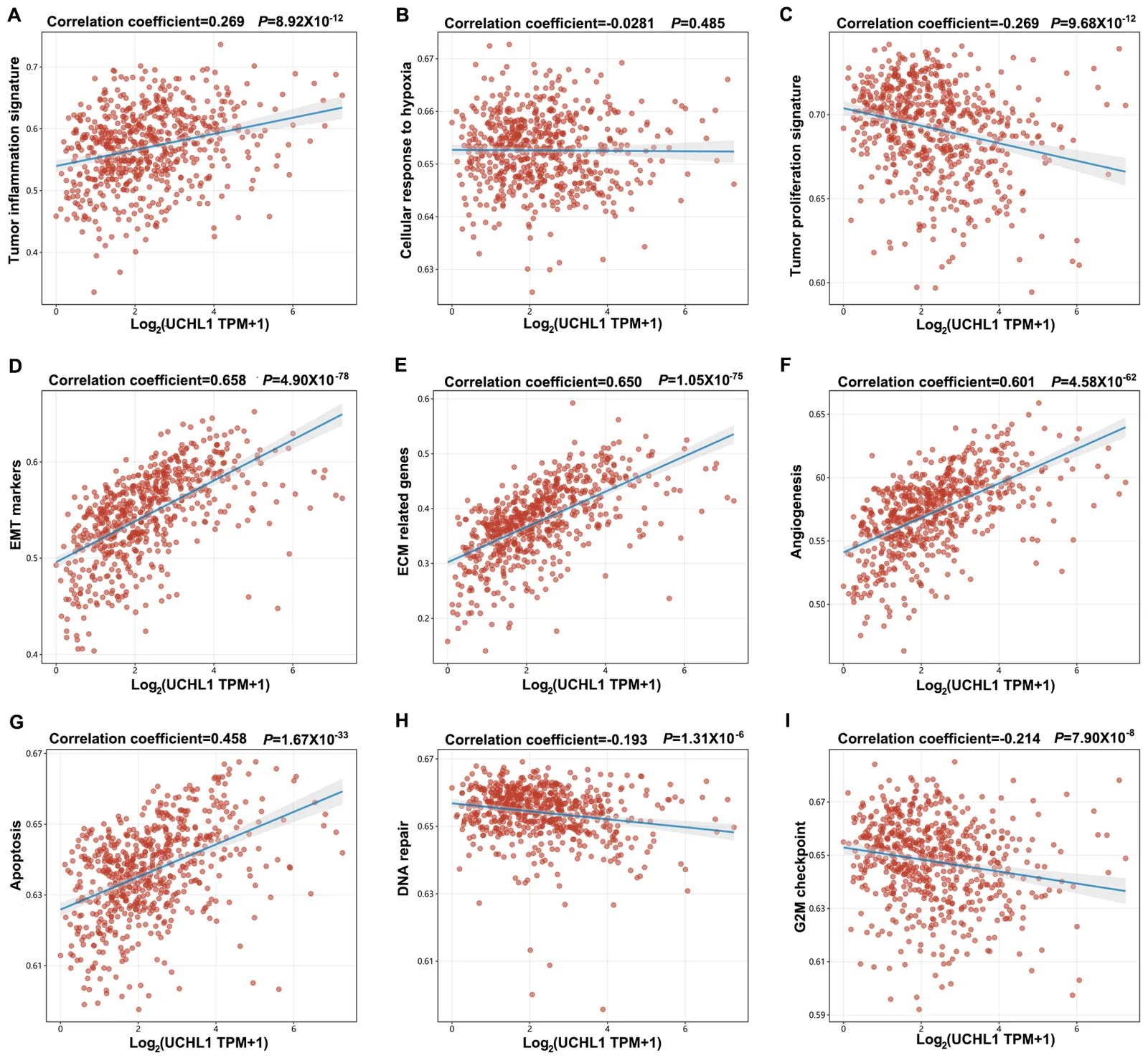
Correlation of *UCHL1* and signaling pathways in CRC. (**A**–**I**) Spearman’s correlation analysis plots reveal the correlation between *UCHL1* expression and tumor inflammation signature (**A**), cellular response to hypoxia (**B**), tumor proliferation signature (**C**), epithelial–mesenchymal transition (EMT) markers (**D**), extracellular matrix (ECM)-related genes (**E**), angiogenesis (**F**), apoptosis (**G**), DNA repair (**H**), and G2M checkpoint regulation (**I**) in CRC samples from The Cancer Genome Atlas database.

Using the ssGSEA algorithm, we calculated the enrichment scores of predefined pathways for each sample to explore the relationship between samples and pathways. The resulting fractions served as an intuitive indicator of sample–pathway interactions. Our findings reveal that *UCHL1* may be involved in several key biological processes in CRC, including tumor inflammation signature, tumor proliferation signature, epithelial–mesenchymal transition (EMT) markers, extracellular matrix (ECM)-related genes, angiogenesis, apoptosis, and G2M checkpoint regulation (Figure 4). Collectively, these results suggest that *UCHL1* plays a multifaceted role in CRC, potentially contributing to tumor progression through the modulation of diverse oncogenic pathways.

### 3.4. The Association Between UCHL1 Expression and Infiltrating Immune Cells

**Figure 5:**
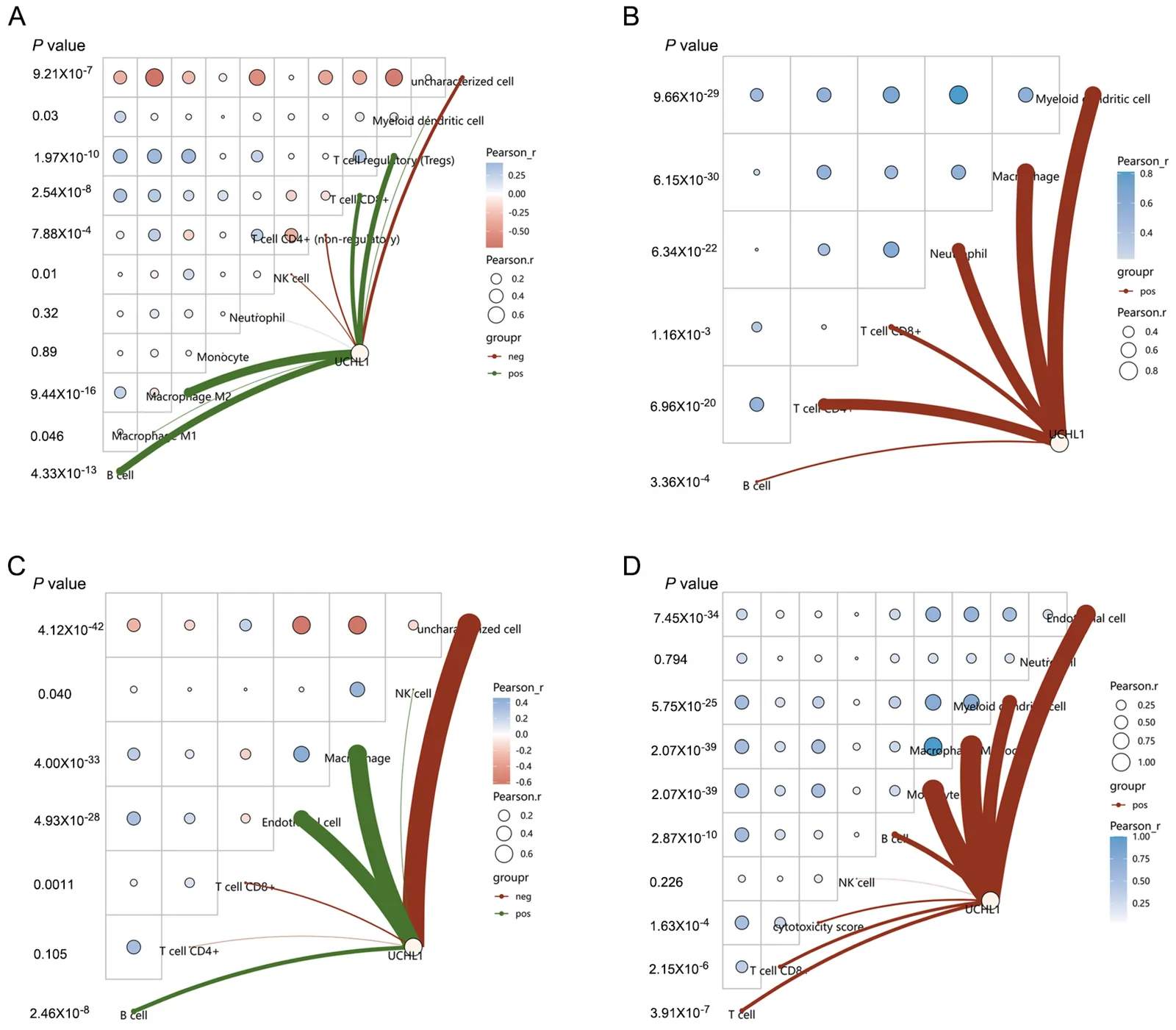
Relationship between *UCHL1* expression and immune scores in CRC: (**A**–**D**) Heatmap illustrates the correlation analysis of *UCHL1* expression with QUANTISEQ (**A**), TIMER (**B**), EPIC (**C**), and MCPCOUNTER (**D**) scores. The redder or greener the color, the greater the correlation between the two; the larger the circle, the stronger the correlation. Pearson correlation analysis was used. All correlations were assessed using Pearson’s correlation analysis.

**Figure 6:**
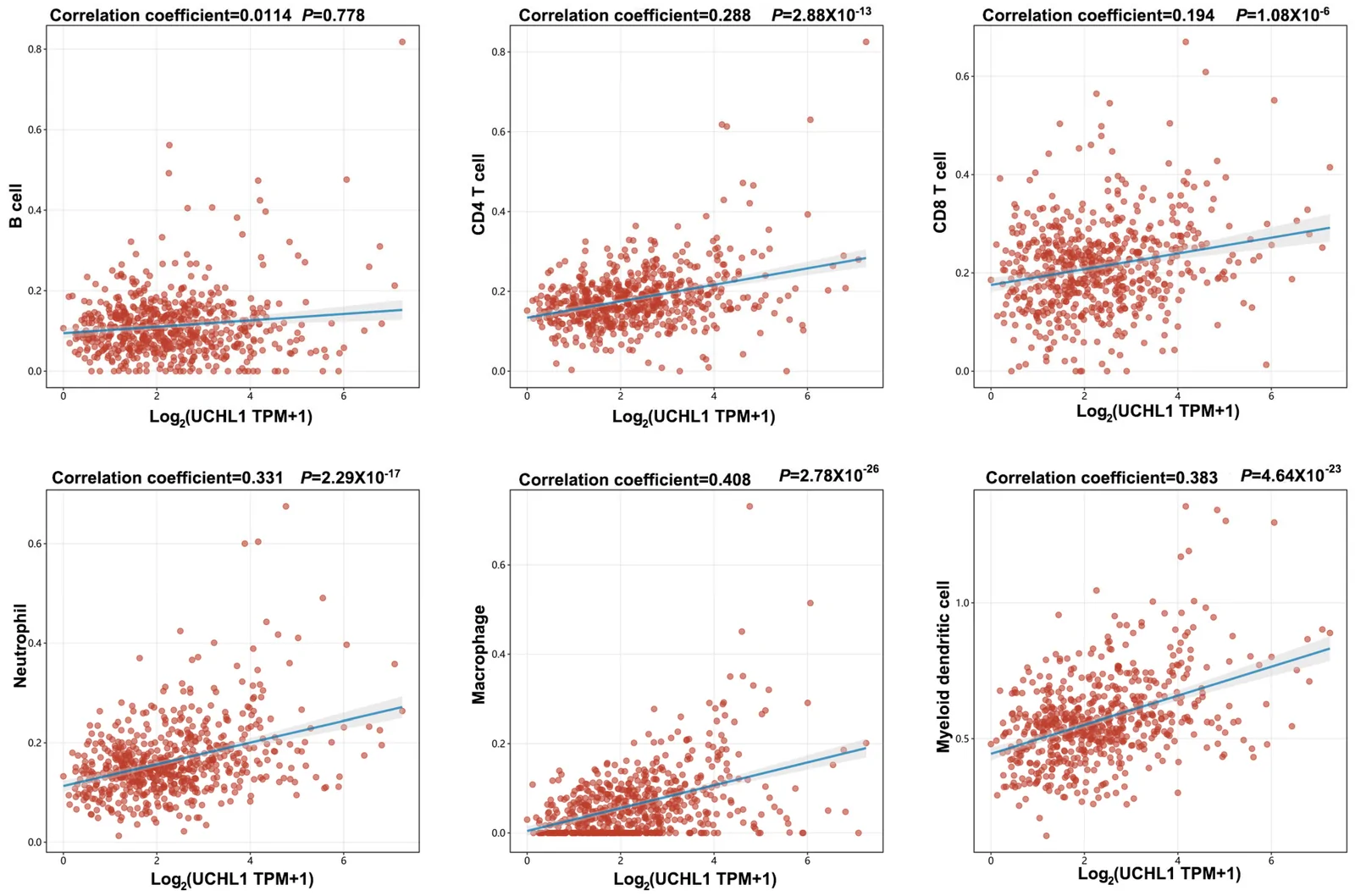
Spearman’s correlation analysis of *UCHL1* expression with immune cells, including B cells, CD4^+^ T cells, CD8^+^ T cells, neutrophils, macrophages, and myeloid dendritic cells in CRC, from The Cancer Genome Atlas database.

To explore the association between *UCHL1* expression and immune cell infiltration in CRC, we conducted a correlation analysis using network diagrams and heatmaps to visualize the relationship between *UCHL1* levels and immune scores. In these plots, color intensity and circle size were used to denote correlation strength, with red and green colors representing negative and positive correlations, respectively. Applying four distinct algorithms—QUANTISEQ, TIMER, EPIC, and MCPCOUNTER—we found that *UCHL1* expression was positively correlated with infiltration of macrophages, CD4^+^ T cells, and CD8^+^ T cells (Figure 5). This positive correlation was subsequently confirmed by Spearman’s rank correlation analysis of TCGA CRC samples via the TIMER database. In agreement with these results, *UCHL1* expression also exhibited positive associations with CD4^+^ T cells, CD8^+^ T cells, macrophages, neutrophils, and myeloid dendritic cells (Figure 6). Collectively, these correlative findings suggest that *UCHL1* expression is associated with the abundance of certain immune cell populations as estimated by computational algorithms.

### 3.5. Relationship Between UCHL1 Expression and Immune Checkpoint Gene Expression

**Figure 7:**
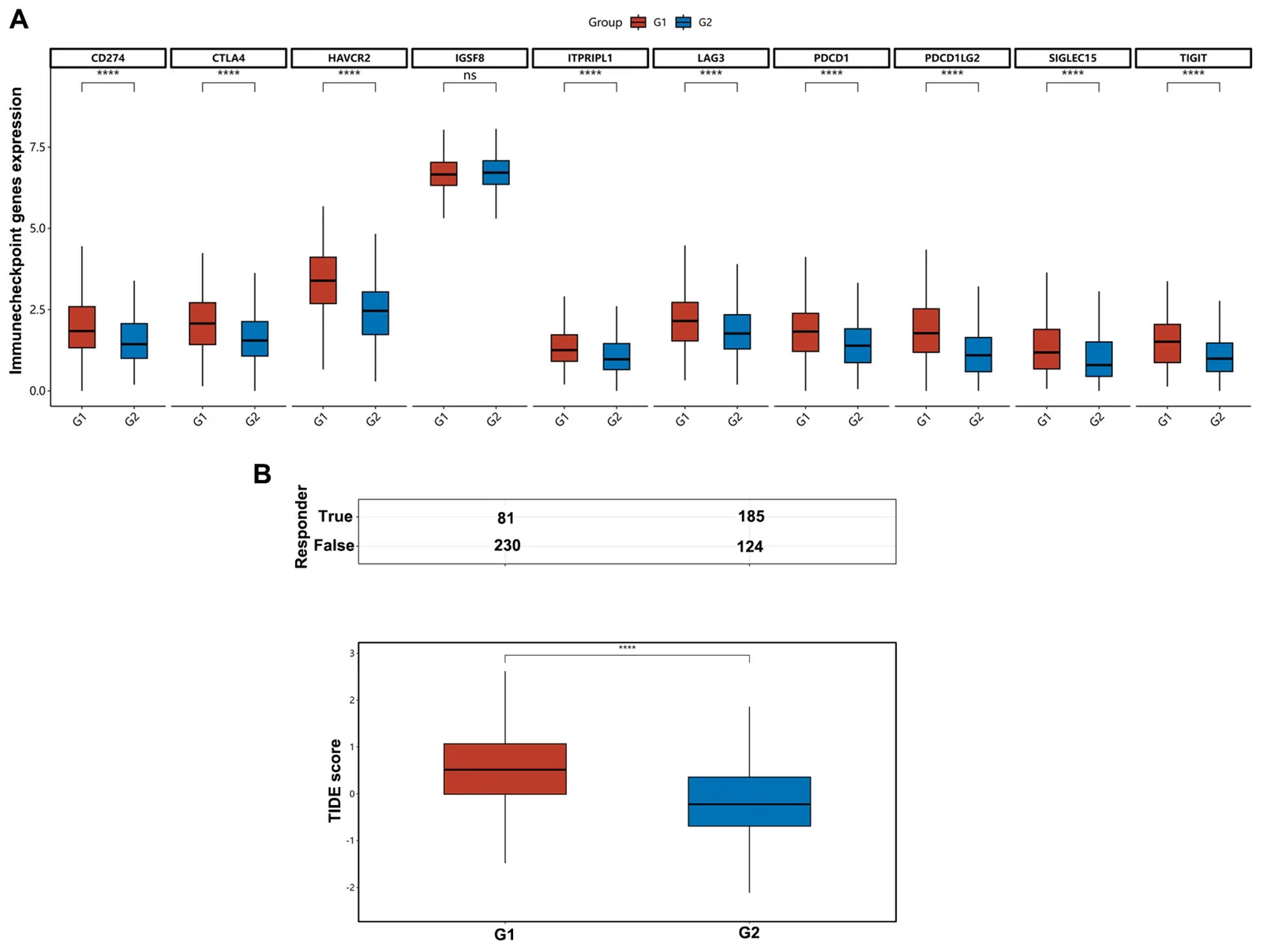
Relationship between *UCHL1* expression and immune response in CRC tissues with high *UCHL1* expression (G1) versus those with low expression (G2): (**A**) A distribution diagram of the expression of immune checkpoint genes in CRC tissues with high *UCHL1* expression (G1) versus those with low expression (G2). (**B**) The box plot of immune response score in CRC tissues with high *UCHL1* expression (G1) versus those with low expression (G2). The significance of differences between the two groups of samples is evaluated by the Wilcoxon rank-sum test. **** *p* < 0.0001; ns, no significant change.

Given the established involvement of *UCHL1* in immunity (Figure 5 and Figure 6), we next investigated its potential association with immune checkpoint genes in CRC. The relationship between *UCHL1* expression and immune checkpoint genes was examined in CRC tissues. As depicted in Figure 7A, the expression levels of several immune checkpoint genes, including *CD274*, *CTLA4*, *HAVCR2*, *ITPRIPL1*, *LAG3*, *PDCD1*, *PDCD1LG2*, *SIGLEC15*, and *TIGIT*, were markedly elevated in CRC tissues with high UCHL1 expression relative to those with low *UCHL1* expression, whereas no significant difference was observed for IGSF8 between the two groups.

To further evaluate the potential clinical relevance of these findings, we employed the TIDE algorithm to predict the immunotherapy response based on *UCHL1* expression levels. The results revealed that CRC patients with high *UCHL1* expression exhibited significantly higher TIDE scores than those with low *UCHL1* expression (Figure 7B).

### 3.6. Protein Expression and Association of UCHL1 with Clinicopathological Variables in CRC Patients

**Figure 8:**
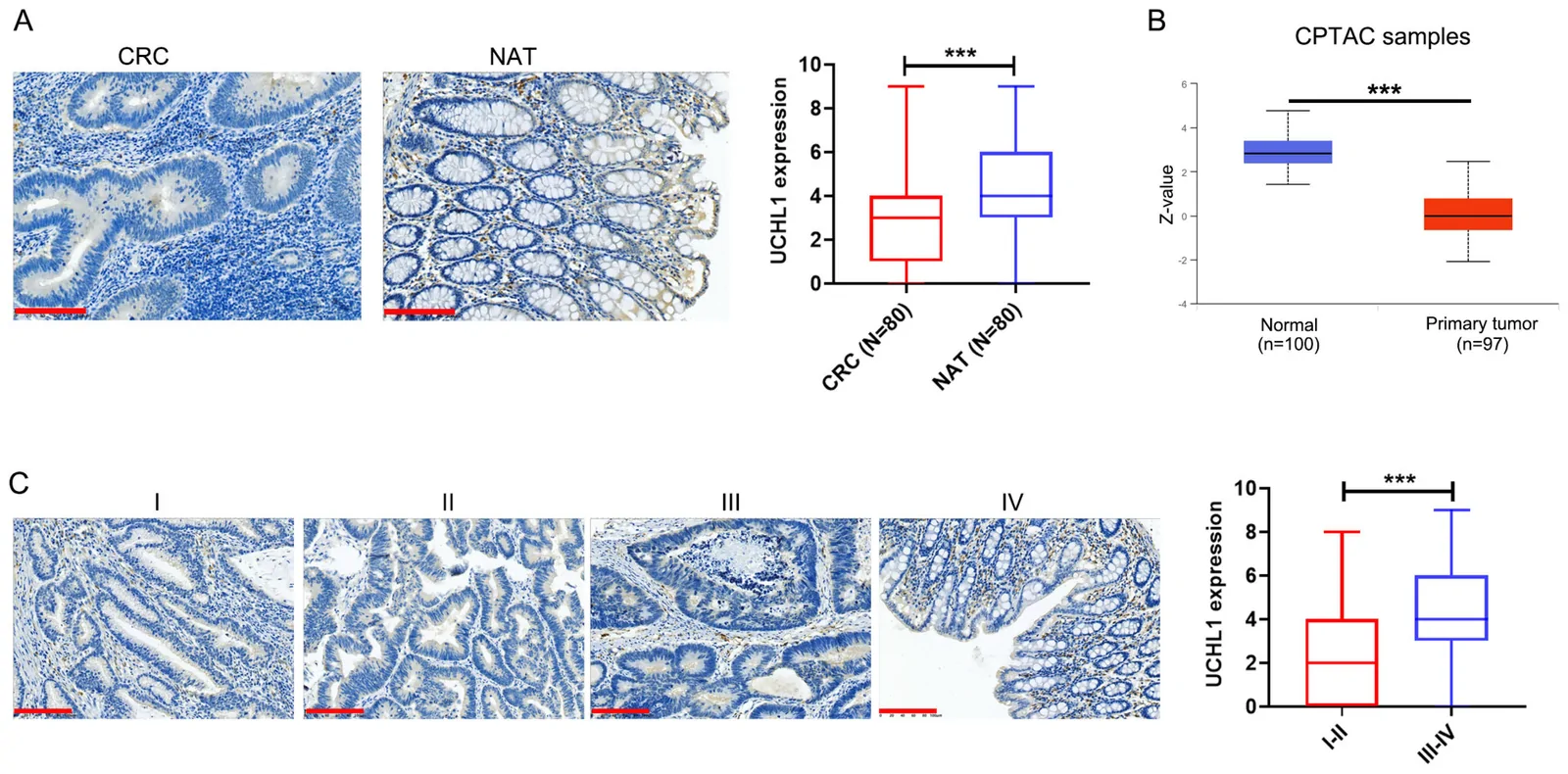
Decreased UCHL1 expression in CRC: (**A**) UCHL1 protein levels in CRC and non-cancerous adjacent tissues (NATs) through immunohistochemistry (IHC). One representative image is shown. Scale bar, 100 µm. (**B**) Protein expression of UCHL1 was significantly decreased in colon cancer tissues compared with that in normal tissues from the CPTAC database. (**C**) The UCHL1 protein expression based on their staining index in CRC specimens at clinical stages I, II, III, and IV. Statistical significance was assessed using an unpaired *t*-test. *** *p* < 0.001.

An IHC analysis was performed to evaluate UCHL1 protein expression in CRC tissues and paired adjacent normal tissues (NATs). The results revealed that UCHL1 expression levels were markedly lower in tumor tissues than in their normal counterparts (Figure 8A). This finding was consistent with data obtained from the CPTAC database (Figure 8B). Additionally, high UCHL1 expression (IHC score > 3) was positively correlated with the N and AJCC stages, whereas no significant association was observed between UCHL1 expression and gender, age, T and M stages, or patient death (Figure 8C, Table 1). Collectively, these findings suggest that reduced UCHL1 expression is a characteristic feature of CRC tissues, while elevated expression may be associated with more advanced disease stages in CRC.

## 4. Discussion

In this study, we comprehensively evaluated the expression, clinicopathological significance, prognostic value, and immunological role of *UCHL1* in CRC through integrative bioinformatics analysis combined with immunohistochemical validation.

Research has demonstrated that UCHL1 expression exhibits tissue-specific patterns across malignancies. For example, in gastric cancer and cervical squamous cell carcinoma, UCHL1 is markedly upregulated in tumor tissues and associated with poor prognosis, supporting its oncogenic role [10,28]. In contrast, aberrant methylation of the *UCHL1* gene has been detected in primary colon cancer samples, suggesting transcriptional silencing [13]. Consistent with this epigenetic observation, our results revealed that *UCHL1* mRNA expression was significantly downregulated in CRC tissues across multiple independent databases, including UALCAN, GEPIA2, GSCA, and ENCORI, as well as in the TCGA cohort. This finding was further validated at the protein level by immunohistochemistry on a tissue microarray, confirming lower *UCHL1* expression in tumor tissues relative to adjacent normal tissues.

Notably, despite its reduced expression in tumors, high *UCHL1* expression was significantly associated with advanced T, N, and TNM stage, as well as with poorer overall survival and disease-free survival in the TCGA and public database analyses. This paradoxical pattern of downregulation in tumor tissues yet correlation with aggressive features has been observed in other malignancies such as ovarian cancer and nasopharyngeal carcinoma, where UCHL1 functions as a tumor suppressor [11,29]. In contrast, UCHL1 promotes tumorigenesis in neuroendocrine carcinomas, triple-negative breast cancer, and gastric cancer [8,9,10], further highlighting its context-dependent role.

To further reconcile this apparent paradox, we performed additional analyses of *UCHL1* promoter methylation using the UALCAN dataset (Figure S1). Our results revealed that the *UCHL1* promoter was hypermethylated in CRC tissues, consistent with previous reports demonstrating that UCHL1 silencing in CRC is primarily driven by CpG island methylation [30,31]. This methylation-expression pattern may partially explain our observation that UCHL1 is globally silenced in the bulk tumor population, yet elevated expression in a subset of aggressive cases correlates with poor prognosis. We speculate that while promoter hypermethylation silences UCHL1 in the majority of tumor cells, selective demethylation, clonal expansion of *UCHL1*-positive aggressive subclones, or epigenetic reprogramming during tumor progression may occur [30]. Additionally, post-transcriptional and post-translational regulatory mechanisms may further uncouple mRNA levels from protein function in advanced stages. For instance, lncRNA ZFAS1 promoted invasion of medullary thyroid carcinoma by enhancing EPAS1 expression via the miR-214-3p/UCHL1 axis [32]. Moreover, S-nitrosylation of the UCHL1 protein itself can modulate its enzymatic stability, deubiquitinating activity [33], and substrate specificity without this modification being reflected by total protein levels measured by IHC. However, as we emphasize throughout this discussion, these mechanistic interpretations remain speculative and are derived solely from correlative bioinformatic and epigenetic analyses.

UCHL1 has been reported to exert multifaceted functions through diverse molecular mechanisms across cancer types. In gastric cancer, reduced UCHL1 expression suppresses proliferation, migration, and invasion, with CIP2A identified as a downstream effector [10]. In triple-negative breast cancer, UCHL1 contributes to endocrine therapy insensitivity by deubiquitinating and stabilizing KLF5 [34]. These studies underscore the functional versatility of UCHL1 in modulating tumor behavior. Consistent with this diversity, our ssGSEA analysis revealed that UCHL1 expression was positively associated with multiple oncogenic processes in CRC, including tumor inflammation signature, tumor proliferation signature, EMT, ECM-related gene expression, angiogenesis, apoptosis, and G2M checkpoint regulation. These findings align with previous reports that UCHL1 promotes CRC progression via activation of the β-catenin/TCF pathway [12]. Collectively, our correlative results extend the functional repertoire of UCHL1 in CRC by implicating it in a broader spectrum of signaling networks beyond previously characterized mechanisms, suggesting that UCHL1 may coordinate multiple oncogenic pathways to drive tumor progression. However, we emphasize that these pathway associations are derived from ssGSEA correlations and do not establish causal relationships.

Given the critical role of the tumor immune microenvironment in CRC progression and therapy response, we investigated the relationship between *UCHL1* expression and immune cell infiltration. Using multiple algorithms (QUANTISEQ, TIMER, EPIC, and MCPCOUNTER), we observed that *UCHL1* expression was positively correlated with computationally estimated infiltration of macrophages, CD4^+^ T cells, and CD8^+^ T cells, a finding consistently validated by Spearman’s correlation analysis. Furthermore, elevated *UCHL1* expression was associated with increased expression of multiple immune checkpoint genes, including *CD274* (*PD-L1*), *CTLA4*, *LAG3*, and *TIGIT*, as well as with higher TIDE scores.

An apparent paradox emerges from our observations: *UCHL1* expression positively correlates with CD8^+^ T cell infiltration, yet also with an immunosuppressive microenvironment and higher TIDE scores indicative of poor immunotherapy response. This seeming contradiction can be reconciled through the framework of T cell dysfunction. The quantity of tumor-infiltrating CD8^+^ T cells does not necessarily equate to their functional quality. In CRC, CD8^+^ T cells that have successfully infiltrated the tumor parenchyma frequently exhibit exhaustion—a state of progressive loss of effector function driven by persistent antigen stimulation and upregulation of inhibitory receptors (PD-1, CTLA-4, and LAG3) [35,36]. Indeed, the TIDE algorithm was specifically designed to model two principal mechanisms of tumor immune evasion, identifying tumors where high CD8^+^ T cell infiltration does not associate with survival benefits. Studies in BRAF V600E-mutant CRC have demonstrated that subtypes with higher CD8^+^ T cell infiltration can exhibit significantly higher T cell dysfunction scores [37].

Emerging evidence directly implicates UCHL1 in suppressing CD8^+^ T cell anti-tumor immunity through multiple mechanisms. In lung adenocarcinoma, UCHL1 deubiquitinates FHL2 to block ferroptosis and counteract CD8^+^ T cell-mediated tumor killing [38]. Additionally, UCHL1 has been shown to promote PD-L1 transcription via the Akt/NF-κB p65 axis [39]. These mechanistic insights align with our correlative observations that UCHL1 expression positively associates with immune checkpoint genes and higher TIDE scores. Collectively, these findings support a model in which *UCHL1*-high CRC tumors recruit CD8^+^ T cells but concurrently suppress their effector function through converging pathways, resulting in a dysfunctional immune microenvironment that fails to control tumor progression and resists immunotherapy.

Based on our correlative observations and emerging evidence from other cancer types—particularly in triple-negative breast cancer, where inhibition of UCHL1 enhances immunotherapy efficacy by stabilizing PD-L1 [40] and where UCHL1 acts as a transporter in the HDAC6/STAT3/PD-L1 pathway [41]—we propose a speculative hypothesis that UCHL1 may potentially contribute to an immunosuppressive microenvironment in CRC, possibly through mechanisms involving immune checkpoint modulation. However, we emphasize that this hypothesis is purely exploratory and derived entirely from bioinformatic correlations; it has not been experimentally validated in CRC and requires direct mechanistic investigation in future studies using CRC cell lines, animal models, and clinical cohorts.

Several important limitations must be acknowledged. First, all pathway enrichment, immune correlation, and TIDE-based predictions presented in this study are derived from descriptive bioinformatic analyses and represent preliminary, hypothesis-generating observations rather than validated mechanisms or clinically actionable biomarkers. Our study lacks functional experiments—such as gain-of-function or loss-of-function assays in CRC cell lines—and in vivo animal models, which are essential to determine whether UCHL1 actively drives immune evasion or whether the observed correlations reflect secondary phenomena. Second, the TIDE algorithm was originally developed and validated using transcriptomic data from patients with melanoma and non-small cell lung cancer who received immune checkpoint blockade therapy [26]. For cancer types outside this training domain, including CRC, TIDE applies a generalized “Other” prediction model whose performance has not been systematically benchmarked against CRC-specific immunotherapy outcomes. Third, our study lacks independent clinical cohorts of CRC patients receiving immune checkpoint inhibitor therapy, which are essential for externally validating the predictive utility of UCHL1 expression. Fourth, TIDE scores are derived from bulk transcriptome data and may not fully capture the spatial heterogeneity of immune cell infiltration and dysfunction within the tumor microenvironment. Future studies incorporating prospective CRC cohorts receiving standardized immunotherapy, along with orthogonal validation methods such as multiplex immunohistochemistry or spatial transcriptomics, are urgently needed to determine whether UCHL1 holds any genuine predictive value in the clinical immunotherapy setting. Despite these limitations, our study provides a comprehensive characterization of UCHL1 expression, clinicopathological associations, and immune correlations in CRC, offering a foundation for future mechanistic and translational investigations.

## 5. Conclusions

In conclusion, this study demonstrates that UCHL1 is downregulated in CRC tissues, but, paradoxically, its elevated expression is associated with advanced disease stages and poor prognosis. UCHL1 is implicated in multiple oncogenic pathways and appears to positively regulate immune cell infiltration and immune checkpoint gene expression, potentially influencing the response to immunotherapy.

## Figures and Tables

**Table 1:** UCHL1 expression and clinical features in 80 colorectal cancer patient samples.

Characteristic	Total No.	UCHL1 Expression		χ^2^	*p* Value
		Low	High		
Gender				0.425	0.5144
male	44	24	20		
female	36	17	19		
Age				3.2241	0.0726
<61	39	24	15		
≥61	41	17	24		
T stage				0.4666	0.4946
T2/T3	38	21	17		
T4	42	20	22		
N stage				14.532	0.0001
N0	46	32	14		
N1/N2	34	9	25		
M stage				1.8139	0.178
M0	68	37	31		
M1	12	4	8		
AJCC stage				12.776	0.0004
I–II	41	29	12		
III–IV	39	12	27		
Death				0.4079	0.523
Alive	58	31	27		
Dead	22	10	12		

## Data Availability

The data of the current study are available from the following open public databases: UALCAN (https://ualcan.path.uab.edu/, accessed on 24 March 2026), GEPIA2 (http://gepia2.cancer-pku.cn/#index, accessed on 24 March 2026), GSCA (https://guolab.wchscu.cn/GSCA/#/, accessed on 24 March 2026), and ENCORI (https://rnasysu.com/encori/, accessed on 24 March 2026) tools.

## Supplementary Materials

The following supporting information can be downloaded at: https://www.mdpi.com/article/10.3390/biomedicines14091924/s1, Figure S1. Methylation of the UCHL1 promoter in COAD and READ based on TCGA datasets from the UALCAN database.

## Abbreviations

CRC	Colorectal cancer
UCHL1	Ubiquitin C-terminal hydrolase L1
IHC	Immunohistochemistry
NATs	Adjacent normal tissues
