# Supplementary material for: UCHL1 Expression in Colorectal Cancer: Clinicopathological Significance, Prognostic Value, and Implications for Immunotherapy Response
Source: Biomedicines. 2026 Aug 27;14(9):1924. doi: 10.3390/biomedicines14091924 (PMC13604906; doi:10.3390/biomedicines14091924)
Supplement: Supplementary file 1 [file biomedicines-14-01924-s001.zip › biomedicines-4509136-supplementary.pdf]

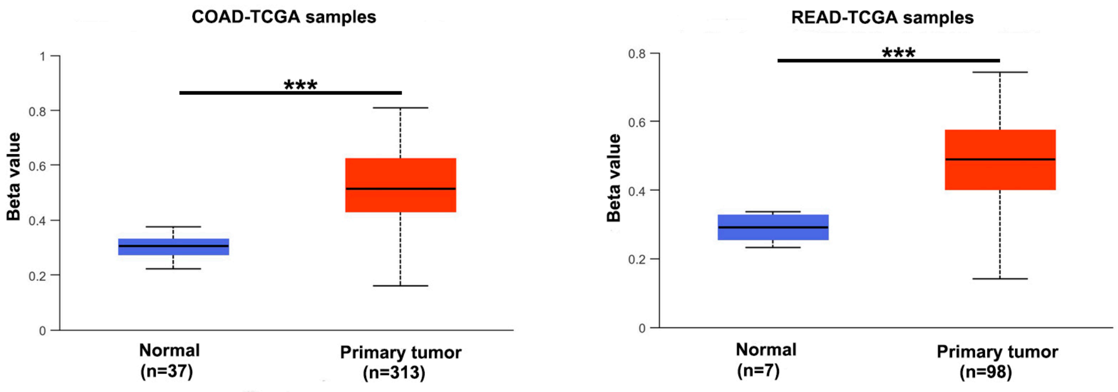

Figure S1. Methylation of the UCHL1 promoter in COAD and READ based on TCGA datasets from the UALCAN database.
